# Physical Activity Habits of Latvian Nursing Students: A Cross-Sectional Study

**DOI:** 10.3390/nursrep12040089

**Published:** 2022-11-30

**Authors:** Una Veseta, Rudīte Lagzdiņa, Maija Rumaka, Lāsma Reide, Voldemārs Arnis, Māra Kampara, Indra Vīnberga, Irēna Upeniece, Maksims Zolovs

**Affiliations:** 1 Department of Sports and Nutrition, Riga Stradins University, LV-1048 Riga, Latvia; 2 Department of Human Physiology and Biochemistry, Riga Stradins University, LV-1048 Riga, Latvia; 3 Statistical Unit, Riga Stradins University, LV-1048 Riga, Latvia; 4 Institute of Life Sciences and Technology, Daugavpils University, LV-5401 Daugavpils, Latvia

**Keywords:** nursing students, physical activity habits, International Physical Activity Questionnaires (IPAQ)

## Abstract

It is important to integrate knowledge about the need for physical activities (PA) in the prevention and care of human health in nursing study programs so that nurses can promote PA among their patients. This study aims to evaluate the PA habits of Latvian nursing students. Participants were students of three universities in Latvia who were enrolled in a four-year bachelor study program, “Nursing”, with a total of 341 after the data cleaning (population size *n* = 1554). The questionnaire contained questions about sociodemographic variables derived from the survey of the Centre for Disease Prevention and Control of Latvia “Health Behaviour among Latvian Adult Population”, habits of PA, and International Physical Activity Questionnaire-Short Form. The data were collected between September and November 2021. Both descriptive and inferential statistics (difference between groups, correlation, and association tests) were calculated to analyse the data. Of the study population, 39% did not reach the minimum WHO-recommended PA. Increased PA level in the later study years is related to more frequent engagement in work and fewer table activities in the curriculum study process, but the higher intensity and total PA could be related to the specifics of nurse and nurse assistant work, which could be especially intense due to the overloaded health care system during the COVID-19 pandemic.

## 1. Introduction

A nurse is a medical person who has completed the basic nursing program and is entitled to work as a nurse in the respective country under the legislation. Nursing care is an integral part of the health care system, which includes health-promoting and disease-preventing measures.

The preamble of the nursing code of ethics in Latvia stipulates that “the main basic tasks of the nursing profession are to promote health, to take preventive measures for health promotion and disease prevention, to help ensure, maintain and restore health, [...]” The code of ethics also stipulates that one of the essential aspects related the professional activity is that “a nurse takes care of his/her health [...]” [1].

An important prerequisite for good health is physical activity (PA), but unfortunately, as mentioned in the report of the World Health Organization, according to the data of “The Health Behaviour of the Latvian adult population study” conducted in 2018, a sufficient level of PA is observed in less than 6% of adults aged 24–54 in Latvia [2]. The “Public Health Assessment” also recognised that PA is not sufficient in the majority of the population; it was found that only 25.4% of the population aged 15–74 practice 30-min-long physical activities at least twice a week [3].

Educating nurses on the need for PA in the prevention and care of human health is especially important in order to integrate knowledge into nursing study programs to provide the future nursing workforce with the skills and confidence they need to promote PA among their patients [4]. In the literature review conducted in 2021, the lack of knowledge about the correct “dosage”, amount, and intensity of physical activities is also mentioned as one of the obstacles preventing nurses from participating in physical activities. It was concluded that it is necessary to promote PA among nurses [5]. A total of 264 nursing students aged 18–30 from four academic years were surveyed at a Spanish university. It was concluded that 68.6% do not practice health-promoting physical activities [6]. Improving the physical fitness of nursing students can enhance their readiness for work and help them to achieve longevity in this physically demanding profession [7]. Nursing students are expected to make healthy lifestyle choices, particularly in terms of PA to improve and maintain their health and well-being. It is important to not only educate nursing students so that they can perform their duties as health promoters in the future but also to be able to take care of their health and follow a healthy lifestyle themselves [8]. Studies show that nursing students experience high levels of stress, anxiety and, depression, which is related to more sitting time. Therefore, PA is an important factor to promote mental health [9]. Promoting PA in nursing students has the potential to increase self-esteem and life satisfaction and decrease the risk of anxiety and depression [10].

Education reform in the nursing profession is currently taking place in Latvia, stipulating that starting from 2022, this profession can only be acquired in a professional Bachelor’s study program. The new model offers one educational process and greater mobility in the labour market. The nurses will also have broader knowledge and competencies after obtaining such education. This reform was conducted under the WHO’s European strategic directions for strengthening nursing and midwifery towards Health 2020 goals [11]. To evaluate the PA habits of Latvian nursing students during their studies, a longitudinal cohort study has been initiated. A cross-sectional study was conducted during the first year and this is the first publication. Every year, using the same research instrument, the same nursing students will be surveyed to determine and analyse the PA habits of Latvian nursing students to provide a reliable research base for policymakers and interested parties.

## 2. Materials and Methods

### 2.1. Study Population 

All participants were students of three universities of Latvia (Riga Stradiņš University, University of Latvia, University of Daugavpils) enrolled in a four-year bachelor study program “Nursing” (*n* = 1554). The sample size was determined as 308 students with a power analysis of a 5% confidence interval and 0.95 confidence level. The sample consisted of 383 nursing students. 

### 2.2. Design 

The recruitment of participants in the cross-sectional study took place between the 27 September and the 26 November 2021 in Latvia. Students were invited to complete an online questionnaire on the data collection platform REDCap. The processing of personal data for this study was upheld following Regulation (EU) 2016/679 of the European Parliament and of the Council on the 27 April 2016.

### 2.3. Measurements 

Study participants filled out a questionnaire, which contained questions derived from the survey of the Centre for Disease Prevention and Control of Latvia “Health Behaviour among the Latvian Adult Population” about sociodemographic variables, including age, gender, education level, income, household arrangement, occupational status, health condition, and PA habits; they also reported their body weight and height. Data on PA during the previous 7 days were collected by the International Physical Activity Questionnaire-Short Form (IPAQ-SF) [12].

From 383 completed questionnaires after IPAQ data cleaning, data from 341 questionnaires were valid for further analysis. Self-reported PA data were managed following the procedure described in the Guidelines for Data Processing and Analysis of the International Physical Activity Questionnaire [13].

The reported time of physical activities in each domain was characterised by the minutes of the metabolic equivalent of the task (MET-minutes) spent on them in one week. Walking MET-minutes were computed as reported walking minutes in one week multiplied by the intensity coefficient 3.3. For moderate and vigorous MET minutes, the coefficients 4.0 and 8.0 respectively were used. A total number of MET minutes/week was derived as a sum of walking, moderate and vigorous MET minutes.

### 2.4. Statistical Data Analysis

The assumption of data distribution was assessed by the Shapiro–Wilk test and an inspection of the normal Q-Q plots. The presence of outliers was inspected according to Hoaglin [14,15] and multivariate outliers according to Cook [16]. The shape of the distribution of cases for all groups of the independent variable was evaluated by boxplots. The Kruskal–Wallis H test was conducted to determine whether there is a significant difference between three or more independent groups. Post hoc analysis was conducted by applying the Dunn test. Spearman’s correlation test was conducted to determine the strength and direction of a linear relationship between two variables (continuous or ordinal). The correlation ratio test was conducted to determine the strength of the association between continuous and multinomial variables. The assumption of adequate cell size was assessed according to Yates, Moore, and McCabe [17]. The chi-square test of homogeneity or Fisher’s exact test was conducted to determine whether differences existed on a dichotomous or multinomial dependent variable between two or more categories of an independent variable.

## 3. Results

A total of 341 nursing students participated. The demographic characteristics of the participants of the study are summarised in Table 1.

More than half of the participants were 18–24 years old. There were more females than males, more single persons than those with other family statuses, most of the participants lived in households without children younger than 18 years and the most common average monthly income for a family member was in the range of 500–1200 EUR. Most of them at the time of the study were students in the 1st and 3rd study year of the Nursing program and were unemployed or full-time workers. The students’ mean age showed a statistically significant correlation with the study year (r_s_ = 0.493, *n* = 341, *p* = 0.001), full-time employment (r_s_ = 0.441, *n* = 341, *p* = 0.001), and the number of children below 18 years of age in the household (r_s_ = 0.324, *n* = 341, *p* = 0.001).

Summarised results of IPAQ-SF are shown in Table 2. Study participants reported the most MET minutes a week were spent in walking activities, but an equal median MET minutes per week were reported in both moderate and vigorous activities.

No differences were found in any activities and sitting times between men and women. 

There were no statistically significant differences between the distribution of MET-minutes a week reported for walking, moderate, high intensity, and in total, between students of different age groups. A Kruskal–Wallis H test showed that there was a statistically significant difference in the sitting time between the different age groups (x^2^(4) = 9.629, *p* = 0.047), with a mean rank sitting time in working days of 178.75 for students in the age group of 18–24 and 137.89 for students in the age group of 35–44 years old. There were no statistically significant differences between the sitting time on days off.

There were statistically significant differences between the mean rank in total MET-minutes a week of 150.43 and 185.18 (x^2^(3) = 8.719, *p* = 0.033) and sitting time in working days of 196.61 and 149.64 (x^2^(3) = 13.800, *p* = 0.003) for students in the 1st and 3rd study years, respectively. 

**Figure 1:**
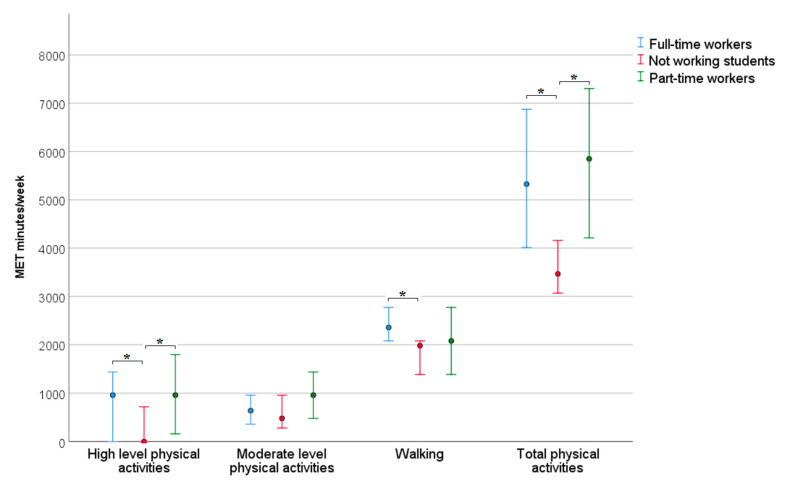
Median and Q1–Q3 of the physical activity of students in different groups according to engagement in work. * *p* < 0.05 indicates a significant difference.

**Figure 2:**
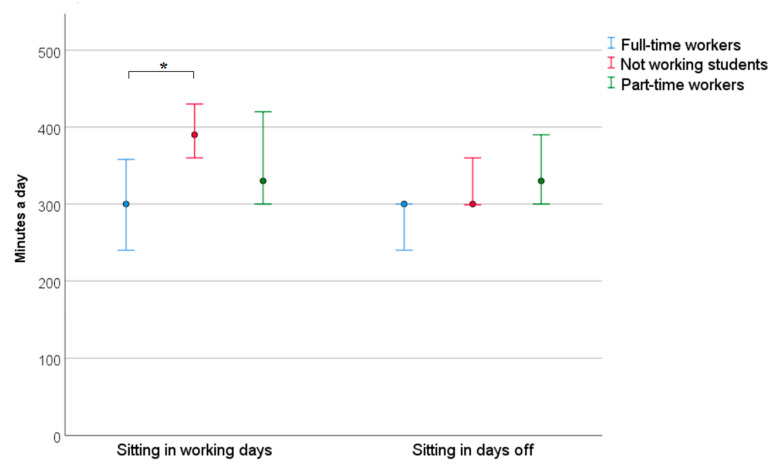
The median and Q1–Q3 of sitting time of students in different groups according to engagement in work. * *p* < 0.05 indicates a significant difference.

Students who were employed in part-time or full-time jobs reported more MET- minutes in total and high-intensity activities a week than students who did not work during the study period. Additionally, full-time employees also presented more MET minutes in walking than the students who did not work (Figure 1). Students who worked full-time jobs reported a lower sitting time on working days, but not on weekends, than students who did not work (Figure 2).

The intensity of PA at work and walking, and cycling minutes to work or university, had a positive association with MET minutes in different activities. In addition, the frequency of engagement in special physical activities of at least 30 min a day in the last 3 months had a positive association with all but the walking MET minutes (Table 3).

There was a negative association between sitting time on a working day and the intensity of PA at work and walking, and cycling minutes to work or university, but sitting time on days off correlated negatively with walking, cycling minutes to work or university and the frequency of engagement in special physical activities of at least 30 min a day in the last 3 months (Table 3). The frequency of engagement in special physical activities of at least 30 min a day in the last 3 months was negatively associated with the intensity of the PA in work (r_s_ = −0.114, *n* = 341, *p* = 0.035).

Of the study population, 39% did not reach the minimum WHO-recommended PA, which stipulates at least 150 min of moderate or at least 75 min of vigorous PA or an equivalent combination of them throughout the week [2]. 

According to the IPAQ scoring protocol [13], 56% of participants were classified as highly active, 30% were classified as moderately active, and 14% were classified as having low PA levels. 

Despite weak associations between the socio-demographic parameters and scores in the different PA domains, each parameter has a minor influence on the PA category according to the IPAQ. The Kruskal–Wallis H test showed that there were no statistically significant differences (*p* > 0.05) in the following parameters: age, study year, current occupational state, income per family member, marital status, education level, and self-evaluation of fitness level, between the low, moderate and high PA category.

Participants in the high or moderate PA level category, in the question, frequency of engagement in special physical activities of at least 30 min a day in the last 3 months, selected the answers 2–3 or 4–6 times per week (x^2^(12) = 32.5, *p* = 0.001, Cramer’s V = 0.218) and about walking or cycling to work/university, the answers were 15–30 or 30–60 min per day (x^2^(8) = 43.6, *p* = 0.001, Cramer’s V = 0.218) more frequently than participants of the low PA category.

## 4. Discussion

This study aimed to determine and analyse PA habits among Latvian nursing students. As far as we know, no research of this kind has been conducted in the population of Latvia before, during, or after the educational reform of the nursing profession in Latvia, but there are many such studies in the world. Global studies emphasise the mission of a nurse as a health promoter, patient and community educator, ambassador of physical activities, and a nurse as such should be an example of a healthy lifestyle. Research is permeated with revelations that the education of nurses in matters of health promotion should be improved, because the full implementation of the nursing mission is not achieved, and one of the effective ways to solve the problem would be improving the education of nurses in matters of health promotion. Therefore, the PA habits of nursing students in Latvia should first be determined to provide a reliable research base for policymakers.

Pre-pandemic studies in other countries show that most nurses have lower levels of daily PA than recommended in the guidelines [18,19,20,21,22,23,24,25]. Professional physical activities of nurses mostly consist of low-intensity physical activities alternated with moderate-intensity tasks [26]. The data of the current study also show that more than a third of the study population did not reach the minimum WHO-recommended PA [27] and a similar study showed that almost half of the nursing students did not reach the WHO-recommended minimum level of physical activity [22].

During the research, the PA of Latvian nursing students could have been affected by the restrictions of the COVID-19 pandemic. From the 11 October 2021, a state of emergency was declared in the country for three months, significantly restricting the unvaccinated. From the 21 October to the 15 November, particularly stringent restrictions for limiting the spread of COVID-19, also known as the “lockdown”, were in effect. A restriction was set to leave the place of residence during the period from 8:00 p.m. to 5:00 a.m., only, providing the opportunity to travel to and from the workplace. During the severe restriction period, no services related to physical activities were provided, and sports events were prohibited, with exceptions for higher-level sports [28]. These restrictions could represent reduced PA during the COVID-19 pandemic, as reflected in other studies. A rapid review conducted by Park and colleagues revealed that mostly overall PA levels fell significantly worldwide during COVID-19 and confinement [29]. This is also supported by the systematic review conducted by López-Valenciano and colleagues specifically on the student population [30]. We cannot assess the changes, because we do not have data with which to compare PA habits of nursing students before COVID-19; however, the results of this study show that more than half of the nursing students involved in the study did high PA. These results regarding the PA of the research participants could also be influenced by the additional work and duties of the working nurses, both in general practitioners’ and inpatient settings, including COVID-19 wards. 

Latvian nursing students were encouraged to become involved in the treatment and care of both COVID-19 patients and other patients. Other students were also involved in providing treatment and care for an ever-increasing flow of COVID-19 patients, as hospitals had exhausted almost all available human resources. During the study, in the autumn of 2021, 761 residents, 544 medical students, and 1310 students in nursing and medical college programs were additionally recruited to Latvian hospitals [31]. The involvement of students in work processes increases their physical activities, as we can see in the results section (Figure 1) and this trend may continue, as there is still a shortage of nurses in Latvia. The work of nurses and nursing assistants involves mostly low and moderate PA during the working process [32,33], which could be the reason for increased PA among working students. However, nursing students who have not yet obtained a nursing education are engaged in work as nursing assistants, which has a higher physical workload than nurses [34].

A higher number of MET minutes spent walking corresponds to the specifics of the work of a nurse/nursing assistant. A study conducted on nurses also observed greater walking activity in nurses [35]. The higher PA and fewer MET minutes spent sitting during working days of working students that we observed in our study may be related to the COVID-19 pandemic in the country at the time, when medical resources were overloaded, increasing the burden on all medical staff, nurses, and nursing assistants in particular. Increased PA at work could lead to less sitting time. At the same time, the educational process during the pandemic was transformed to remote learning using computers, which increased sitting time and reduced walking time for students who were not working, as there was no longer a need to commute to and from the university and their place of residence.

According to the IPAQ assessment protocol [13], the results of our study show that approximately half of the participants were classified as expressly active, almost a third—as moderately active, and the rest of the students were classified as leading a low-activity lifestyle. Such a result could be explained by the fact that most of the students were in clinical practice during the data collection period, which involved both commuting to the place of practice and physically hard work at the place of practice. This is also reflected in the results collected in the study, which show a positive correlation of total PA with commuting to the work/educational institution on foot or by bicycle, as well as physical intensity at work. These two factors (intensity of PA at work and walking/cycling to university) have a positive correlation with the minutes of all activities evaluated by the IPAQ—walking, moderate, vigorous and as a result also with the total amount of MET-minutes). In a study in which the physical activity levels of the nursing students were measured over time, in three semesters, the students generally maintained high levels of physical activity during their studies, which did not change statistically. The predominant activity was walking, followed by vigorous activity and then moderate activity [36].

Although most studies still conclude that PA among medical care students, nursing students included, is insufficient, some studies show similar results and conclude that more than half of nursing students show a high level of PA [37]. The results of our study on higher PA rates for those participants who walk or cycle to the workplace or the university more often are consistent with the findings of a Finnish study showing that individuals who are active outside of work engage in more specialised and organised physical activities, are more active in their commuting habits to/from the workplace, and have a higher tendency to participate in activities at work. The study also found that these tendencies were observed in women twice as much as in men under the same transport and infrastructure conditions [38]. This aspect could be significant in promoting PA among nursing students.

Our study showed that walking is the predominant PA of nursing students, which is similar to the studies conducted by American and Polish authors in this population [36,39].

According to the WHO recommendations [27], adults aged 18–64 years should do at least 150–300 min of moderate-intensity aerobic physical activity; or at least 75–150 min of vigorous-intensity aerobic physical activity; or an equivalent combination of moderate- and vigorous-intensity activity throughout the week.

These recommendations indicate that the intensity of the exercise should be measured on a scale from 0 to 10 relating to the individual’s abilities. Low intensity refers to PA performed at an intensity from 3 to 6 times the resting intensity, moderate-intensity PA is usually 5 to 6, and high intensity refers to PA performed at 6.0 or more METs, or about the individual’s abilities, which is 7 or 8 on a scale of 0 to 10 [27]. Therefore, PA experts also recommend evaluating intensity not only by absolute indicators (METs) but by relative indicators depending on physical fitness [40]. For a physically untrained person, 6 METs can be an exercise that is already close to the maximum possible intensity, but for a well-trained person, it is an exercise of low intensity.

In a meta-analysis, which reviewed the currently available studies in which technology was used to evaluate PA, it was found that physical activities of various intensities play a role in reducing morbidity and all-cause mortality; however, low-intensity exercise (1.5–2.9 METs) only reduces the risks of morbidity and mortality if a very high amount of activity is provided, i.e., physical activity for many hours every day, reaching the maximum risk reduction with physical activity that lasts more than 6 h a day. In contrast, moderate-intensity PA (5–6 METs) maximally reduces the risk (by 60%) at 80 min of activity per day, and high-intensity PA at 24 min per day [41]. The idea is expressed that heavy PA at work does not yet guarantee that it is healthy and it is likely that, depending on the nature of the work, the levels required for health benefits may also vary between 150 and 300 min of moderate to vigorous intensity PA (MVPA). For nurses, as healthcare workers, if PA at work is lower than the average intensity load, it is recommended to add 150 min/week of MVPA as leisure time and transport activity [42].

This study’s shortcomings include the electronic survey designed to be filled in by each participant individually, which was chosen for data collection. This tool was chosen because direct contact had to be reduced due to COVID-19 restrictions. In a systematic review conducted in 2011, 23 studies comparing the instruments for determining the level of PA, the International Physical Activity Questionnaire Short Form (IPAQ-SF), and one of the objective methods of measuring PA were analysed. Their conclusions show that the indicators of PA collected by the IPAQ-SF are significantly overestimated and their reliability is weak, so it is important to correctly interpret the results and, if possible, use an additional objective tool for determining the level of PA [43]. COVID-19 restrictions and the evaluation of our technical capabilities prevented us from considering the use of more objective methods. Since the study is cross-sectional, we cannot determine the exact cause-effect relationships. We are looking forward to seeing longitudinal data in the next few years to determine student physical activity changes over the study period.

Our study has several strengths. This is the first study to analyse the PA habits of Latvian nursing students. Secondly, a sufficiently large group of nursing students in every year of study was covered, so the results can be attributed to this entire population. Thirdly, the electronic survey designed to be filled in individually by each participant made it possible to reach a variety of students, not only those who are positive about physical activity. Fourthly, half of the respondents agreed to complete the same questionnaire a year from now, thus, the study will be continued and the changes in PA during the study will be analysed, and consequently, our work will provide a reliable research base for policymakers and interested parties to support appropriate organisational interventions, such as introducing educational activities in the study program and places of practice to motivate and promote positive action in the field of health. Physical activities could be a good alternative to some unhealthy stress-reduction solutions (smoking, emotional eating, etc.).

## 5. Conclusions

An increased PA level in the later study years (study year 3 vs. 1) is related to more frequent engagement in jobs and fewer table activities in the curriculum. The higher intensity and total physical activities of full and part-time working nursing students’ could be related to the specifics of nurse and nurse assistant work, which could be especially intense due to the overloaded healthcare system during the COVID-19 pandemic, as well as travel to and from work. Most of the students (61%) reach the WHO minimum recommended PA, and 86% are classified as highly or moderately active according to the IPAQ-SF data. Students who engage in special physical activities for at least 30 min a day at least twice a week, or who walk or cycle to work/university for longer than 15 min, have a greater probability of reaching a high PA level according to the IPAQ-SF.

Further investigation should be conducted to determine the long-term effects of the COVID-19 pandemic on the PA of nursing students.

## Figures and Tables

**Table 1:** Demographic characteristics of the study participants.

Variables	Frequency	Percent
Sex		
Female	330	96.8
Male	11	3.2
Age (years)		
18–24	195	57.2
25–34	52	15.2
35–44	61	17.9
45 and more	33	9.7
Study year		
First	121	35.5
Second	68	19.9
Third	105	30.8
Fourth	47	13.8
Employment status (in addition to the studies)		
Unemployed	127	37.2
Part-time employee (working less than 40 h/week)	87	25.5
Full-time employee (working 40 h/week)	127	37.2
Single	183	53.7
Married or domestic partnership	139	40.8
Divorced, widowed, or separated	19	5.6
Income for a family member in a month		
Below EUR 500	103	30.2
EUR 500–1200	169	49.6
Above EUR 1201	69	20.2
Number of children below 18 years of age in the household		
0	182	53.4
1	84	24.6
2 and more	75	22.0

**Table 2:** Characteristics of PA assessed by IPAQ.

Parameter	Median	Interquartile Range (Q1–Q3)
Walking (MET-min/week)	2079	3145 (990–4134.9)
Moderate PA (MET-min/week)	720	1800 (0–1800)
Vigorous PA (MET-min/week)	720	2480 (0–2480)
Total PA (MET-min/week)	4266	6788 (2238–9025.5)
Sitting on a working day (min/day)	360	241(239.5–480)
Sitting on a day off (min/day)	300	300 (180–480)

**Table 3:** Correlation coefficients between IPAQ domains and self-evaluation of PA habits.

	Walking (MET-min/Week)	Moderate PA (MET-min/Week)	Vigorous PA (MET-min/Week)	Total PA (MET-min/Week)	Sitting on a Working Day (min/Day)	Sitting on a Day off (min/Day)
The intensity of PA at work	0.176 **	0.244 **	0.226 **	0.337 **	−0.301 **	
Walking or cycling to work/university (min/day)	0.261 *	0.154 *	0.140 *	0.247 **	−0.141 *	−0.119 *
The frequency of engagement in special physical activities of at least 30 min a day in the last 3 months		0.220 **	0.172 **	0.128 *		−0.116 *

* *p* < 0.05, ** *p* < 0.001 (Spearman’s correlation test).

## Data Availability

The datasets analysed during the current study are not publicly available yet because this data will be used to prepare at least one additional publication after which data will be publicly available. Currently, data are available from the corresponding author on reasonable request.
